# Data on floating treatment wetland aided nutrient removal from agricultural runoff using two wetland species

**DOI:** 10.1016/j.dib.2018.12.037

**Published:** 2018-12-15

**Authors:** Jonathan T. Spangler, David J. Sample, Laurie J. Fox, James S. Owen, Sarah A. White

**Affiliations:** a Department of Biological System Engineering, Hampton Roads Agricultural Research and Extension Center, Virginia Polytechnic and State University, Virginia Beach, VA 23455, USA; b School of Plant and Environmental Sciences, Hampton Roads Agricultural Research and Extension Center, Virginia Polytechnic and State University, Virginia Beach, VA 23455, USA; c School of Agricultural, Forest, and Environmental Sciences, Clemson University, E-143 Poole Agric. Center, Clemson, SC 29634-0310, USA

## Abstract

The data presented in this article are related to the research article entitled “Floating treatment wetland aided nutrient removal from agricultural runoff using two wetland species” (Spangler et al., 2018). This Data in Brief article provides data on concentrations of common ions, macro- and micro-nutrients and metals every other week during a floating treatment wetland (FTW) mesocosm experiment, and macro- and micro-nutrient contents in cumulative plant tissues, data on continuously monitored water temperature, and nitrogen and phosphorus removal curves assessed every other week. The full data set is made available to enable critical or extended analysis of the research.

**Specifications table**

**Table:** 

Subject area	*Ecological engineering*
More specific subject area	*Phytoremediation*
Type of data	*Figures, tables*
How data were acquired	*Field study from replicated mesocosms*
Data format	*Summary, analyzed*
Experimental factors	*Nutrient concentration, plant species*
Experimental features	*Replicated mesocosms*
Data source location	*Country: United States of America*
Data accessibility	*The data are available with this article.*

**Value of the data**

• The data provide macro- and micro-nutrient data on the FTWs evaluated.
• The data provide temperature data on the FTW treatments evaluated.
• The data provide fitted weekly curves for N and P removal enabling assessments of FTW nutrient uptake rate variation across the growing season.
• The data enable additional analysis of the same FTW experiment.

## 1 Data

Floating treatment wetlands (FTWs) are an emerging treatment technique that may enhance water quality treatment of retention ponds by increasing removal of nutrients, namely nitrogen (N) and phosphorus (P). FTWs float on rafts in a water body on which wetland plants are planted [2]. Nutrients are removed through uptake into plant tissue, biochemical reactions that drive off nitrogen (N_2_) gas, and settling of precipitants or sorbed complexes within the water column [3]. Assessments of floating treatment wetlands (FTWs) can be made using large-scale field studies, or in smaller, mesocosm scale experiments. Mesocosms provide several advantages, one of which is they can be replicated easily, increasing the power of the experiments by incorporating variability. The mesocosm approach facilitates collection of key variables that may be related to performance [4].

Effluent from simulated open-air nursery runoff with high nutrient concentrations (17.1 mg L^−1^ total-N or TN and 2.61 mg L^−1^ total-P or TP) and urban runoff with low nutrient concentrations (5.22 mg L^−1^ TN and 0.52 mg L^−1^ TP) were run through parallel, replicated mesocosms over a 19-week growing season. Treatments included two plants, *Pontederia cordata*, *Juncus effuses*, a raft with no plants, and a mesocosm with no raft (open-water), the latter two as controls. Performance was evaluated based upon a 7-day hydraulic retention time.

**Fig. 1:**
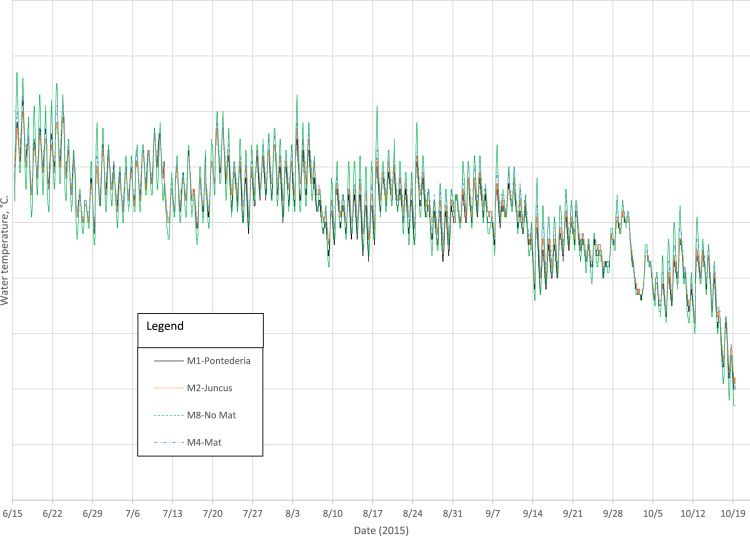
Water temperature during the experiment for selected treatments.

**Fig. 2:**
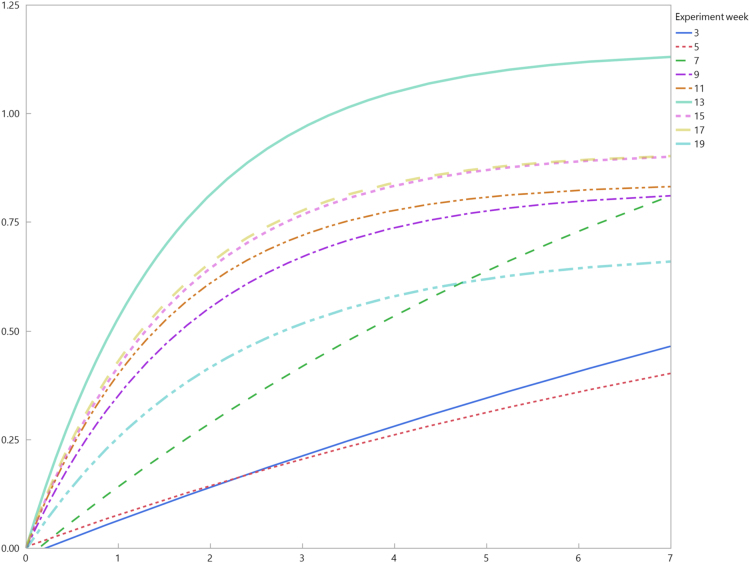
Example of a weekly nutrient removal curve for TP removal curves for high initial concentration (17.13 ± 0.24 mg L^−1^ TN and 2.61 ± 0.04 mg L^−1^ TP) *Pontederia cordata* treatments from June 2015 through October 2015.

Summary mesocosm water quality data for ionic species and elemental analysis are provided in Appendix A, Tables A-1 through A-7. Summary composite mesocosm plant tissue results are provided in Appendix B, Tables B-1 and B-2. Hourly water temperature data for a single mesocosm for each of the four treatments are shown Fig. 1; daily minimum and maximum water temperatures are provided in Appendix C, Table C-1. An example of a weekly nutrient removal curve is shown in Fig. 2. A complete set of these figures is provided in Appendix D, Figs. D-1 through D-8. Statistical summaries of the fitted curves are provided in Appendix D, Tables D-1 through D-16.

## 2 Experimental design, materials, and methods

### 2.1 Location

Data were collected at the Virginia Tech Hampton Roads Agriculture Research and Extension Center (HRAREC; 36°53′ N, 76°10′ W), located in northwest Virginia Beach. During the study, measured precipitation was 559 mm and the mean, minimum and maximum air temperatures were 24.7, 7.5, and 36.3 °C. Thirty-two structural foam stock tanks (Rubbermaid Commercial Products, Winchester, VA, USA) with a capacity of 378.5 L were used as mesocosms; each was retrofitted with an overflow that kept the water volume to a maximum of 302.8 L.

### 2.2 Experimental design

The experiment was set up in a randomized complete block design that included four mesocosm replicates of eight treatments (Table 1). High and Low nutrient concentrations were mixed using synthetic fertilizer to achieve a concentration in the high (17.1 mg L^−1^ TN and 2.61 mg L^−1^ TP) and low (5.22 mg L^−1^ TN and 0.52 mg L^−1^ TP) concentration ranges. Simulated runoff was created by adding water and 66.2 g (low concentration) or 368.0 g (high concentration) water soluble fertilizer (24-8-16 Southern Agriculture Insecticides Inc., Hendersonville, NC, USA) to each mix tank. Plants were purchased from Environmental Concerns (St. Michaels, MD, USA) and were approximately 5 cm in length (plugs) when planted.

**Table 1:** Treatment combinations for the FTW wetland study.

**Treatment**	**Mat**	**Plants**	**Species**	**Concentration**
1	Yes	Yes	*Pontederia cordata*	Low
2	Yes	Yes	*Pontederia cordata*	High
3	Yes	Yes	*Juncus effusus*	Low
4	Yes	Yes	*Juncus effusus*	High
5	Yes	No	n/a	Low
6	Yes	No	n/a	High
7	No	No	n/a	Low
8	No	No	n/a	High

The water depth in each mesocosm was 47 cm when full, with a surface area of 0.79 m^2^. The rafts were manufactured by Beemats (Beemats LLC, New Smyrna Beach, FL, USA) using a 1.3 cm thick closed cell foam. Mats were trimmed to fit the mesocosms leaving a total mat surface area of 0.64 m^2^ (0.55 m^2^ if the precut holes are subtracted). This resulted in approximately 80.3% of the water surface being occupied by the raft.

Prior to planting, the roots of each plant were rinsed to remove as much of the original planting media as possible. Immediately after rinsing, the roots were wrapped in coir, placed in the plastic aerator cups, both obtained from Beemats (Beemats LLC, New Smyrna Beach, FL, USA), transported to the site, and planted. Each mat contained 20 planting holes; 50% of which were used. Five plants were randomly planted on each half of the mat for a total of ten plants per mesocosm (approximately 15 plants/m^2^). After each 7-day hydraulic residence time (HRT), the mesocosms were drained and refilled with a new batch of simulated runoff. During the first five weeks, the high concentration treatment only received 221 g (10.6 mg L^−1^ TN and 1.68 mg L^−1^ TP) of fertilizer to allow for plant acclimation.

### 2.3 Water sampling and analysis

The start of each experimental week (7-day HRT) was designated as day 0. On day 0, grab samples were collected into 125 mL wide-mouth Nalgene bottles (Thermo Fisher Scientific, PA, USA) from each mix tank at approximate 30 cm (12 in.) below the water surface after each mesocosm was filled with low or high nutrient solution to represent the initial nutrient concentration for the 7-day HRT. The start of each experimental week was also day 7 of the prior 7-day HRT. On day 7, 125 mL grab samples were collected from each of the 32 mesocosms prior to draining. Additionally, water temperature, pH, DO, and EC measurements were taken *in situ* at a depth of 30 cm. These samples reflected the final (post-treatment) nutrient concentrations for the retention period. Additionally, grab samples and in situ measurements were taken at the 30 cm depth every other week on days 3 and 5 for each mesocosm.

After collection, all water samples were kept on ice in a cooler until other necessary fieldwork was complete. Subsamples were frozen until analysis could be performed or were acidified to a pH of 2 in preparation for shipping. All collected water samples were analyzed at Virginia Tech for TN and TP using automated flow injection analysis after persulfate digestion (persulfate digestion methods QuickChem® Method 10-107-04-4B and 10-115-01-4B; Lachat Instruments, Loveland, CO, USA). In addition, water samples were analyzed for metal content using inductively coupled plasma optical emission spectrometry (ICP-OES 7400 Duo, Thermo Fisher Scientific, PA, USA).

### 2.4 Plant tissue sampling and analysis

Tissue samples were collected from three plants chosen at random from each mesocosm after 19 weeks. Immediately following the harvest, the roots and shoots were separated and stored in paper bags. The roots and shoots from *P. cordata* samples were separated at the crown of the plant. The roots and shoots from *J. effusus* samples were separated at the bottom of the planting cup and the crown on the plant, respectively. This procedure was modified slightly to accommodate *J. effusus*, as the portion of roots in the planting cups was too entangled with the planting coir for complete separation. The samples were dried in a forced air oven at 58 °C until all moisture was released and consistent sample weights were maintained for two consecutive days. Tissue samples were then ground to ≤0.5 mm particle size using a 3379-K35 Variable Speed Digital ED-5 Wiley Mill set to 900 RPM (Thomas Scientific, Swedesboro, NJ, USA). A single composite sample was made from the three plants and was thoroughly mixed. The same procedure was used for the roots. Tissue samples were analyzed. Samples were analyzed for TN in a nitrogen combustion analyzer (LECO FP528 Nitrogen Combustion Analyzer, Leco Corp., St. Joseph, MI, USA). Samples were also analyzed for TP on an inductively coupled plasma-optical emission spectrometer (Spectro Arcos ICP-OES, Spectro Analytical Instruments-a division of Ametek, Kleve, Germany).

### 2.5 Data analysis

Water sample data were reported with concentration units (mg L^−1^) and was converted to mass using mesocosm volume. Mesocosm volume was tracked using calculated evapotranspiration and a water balance model described in the main paper [1].

## Appendix A Supplementary material

Summary water quality data of mesocosm experiment.

Summary plant tissue data of mesocosm experiment.

Summary water temperature data.

Nitrogen and phosphorus weekly removal curves. Figure D-1. Weekly TP removal curves for high initial concentration (17.13 ± 0.24 mg L^−1^ TN and 2.61 ± 0.04 mg L^−1^ TP) *Pontederia cordata* treatments from June 2015 through October 2015. Figure D-2. Weekly fitted TN removal curves for high initial concentration (17.13 ± 0.24 mg L^−1^ TN and 2.61 ± 0.04 mg L^−1^ TP) *Juncus effusus* treatments from June 2015 through October 2015. Figure D-3. Weekly fitted TP removal curves for high initial concentration (17.13 ± 0.24 mg L^−1^ TN and 2.61 ± 0.04 mg L^−1^ TP) *Juncus effusus* treatments from June 2015 through October 2015. Figure D-4. Weekly fitted TN removal curves for low initial concentration (5.22 mg L^−1^ TN and 0.52 mg L^−1^ TP) *Pontederia cordata* treatments from June 2015 through October 2015. Figure D-5. Weekly fitted TP removal curves for low initial concentration (5.22 mg L^−1^ TN and 0.52 mg L^−1^ TP) *Pontederia cordata* treatments from June 2015 through October 2015. Figure D-6. Weekly fitted TN removal curves for low initial concentration (5.22 mg L^−1^ TN and 0.52 mg L^−1^ TP) *Juncus effusus* treatments from June 2015 through October 2015. Figure D-7. Weekly fitted TP removal curves for low initial concentration (5.22 mg L^−1^ TN and 0.52 mg L^−1^ TP) *Juncus effusus* treatments from June 2015 through October 2015. Figure D-8. Weekly fitted TN removal curves by day for high initial concentration (17.13 ± 0.24 mg L^−1^ TN and 2.61 ± 0.04 mg L^−1^ TP) *Pontederia cordata* treatments from June 2015 through October 2015.
