# Supplementary material for: Data on floating treatment wetland aided nutrient removal from agricultural runoff using two wetland species
Source: Data Brief. 2018 Dec 15;22:756–61. doi: 10.1016/j.dib.2018.12.037 (PMC6330358; doi:10.1016/j.dib.2018.12.037)
Supplement: Supplementary file 2 — Summary water quality data of mesocosm experiment. [file mmc2.zip › Table A-6.docx]

**Table A-6.** Ion species concentration for nitrate, ammonium, nitrite, and phosphate at day 7 of 5 of 19 weeks.

| **Exper-** | **Presence or** | **Mat, No** |  | | |  |  |  |  |  |  |  |  |  |
| --- | --- | --- | --- | --- | --- | --- | --- | --- | --- | --- | --- | --- | --- | --- |
| **Mental** | **absence** | **mat/taxa** |  |  |  |  |  |  |  |  |  |  |  |  |
| **Week No.** | **of plants** | **planted** |  |  |  |  |  |  |  |  |  |  |  |  |
|  |  | **within mat** | **Nitrate (mg/L)** | | | **Ammonium (mg/L)** | | | **Nitrite (mg/L)** | | | **Phosphate (mg/L)** | | |
| 3 | No Plant | Mat | 11.52 | ± | 1.31 | 1.86 | ± | 0.33 | BD | ± | BD | 2.22 | ± | 0.66 |
|  |  | No Mat | 4.68 | ± | 1.41 | 1.08 | ± | 0.27 | 0.22 | ± | BD | 1.78 | ± | 0.38 |
|  | Plant + Mat | Juncus | 10.43 | ± | 1.19 | 2.04 | ± | 0.38 | BD | ± | BD | 2.46 | ± | 0.75 |
|  |  | Pontederia | 5.39 | ± | 1.38 | 0.46 | ± | 0.22 | BD | ± | BD | 1.94 | ± | 0.20 |
| 7 | No Plant | Mat | 19.69 | ± | 4.26 | 3.04 | ± | 0.24 | 1.89 | ± | 1.52 | 4.83 | ± | 1.46 |
|  |  | No Mat | 8.58 | ± | 2.49 | 4.55 | ± | 0.26 | 1.16 | ± | 0.82 | 5.38 | ± | 0.00 |
|  | Plant + Mat | Juncus | 14.18 | ± | 4.62 | 2.71 | ± | 0.37 | 0.80 | ± | 0.00 | 5.41 | ± | 1.62 |
|  |  | Pontederia | 0.36 | ± | 0.00 | BD | ± | BD | BD | ± | BD | 0.23 | ± | 0.00 |
| 11 | No Plant | Mat | 12.73 | ± | 3.77 | 3.74 | ± | 0.05 | 1.77 | ± | 0.58 | 4.10 | ± | 1.29 |
|  |  | No Mat | 15.00 | ± | 3.97 | 4.88 | ± | 0.09 | 3.58 | ± | 0.50 | 6.09 | ± | 0.60 |
|  | Plant + Mat | Juncus | 20.48 | ± | 5.75 | 2.43 | ± | 0.09 | BD | ± | BD | 5.06 | ± | 0.51 |
|  |  | Pontederia | BD | ± | BD | BD | ± | BD | BD | ± | BD | BD | ± | BD |
| 15 | No Plant | Mat | 23.50 | ± | 5.43 | 5.90 | ± | 0.03 | 2.24 | ± | 0.43 | 4.48 | ± | 1.31 |
|  |  | No Mat | 11.44 | ± | 3.94 | 5.58 | ± | 0.02 | 3.75 | ± | 1.89 | 6.10 | ± | 0.93 |
|  | Plant + Mat | Juncus | 22.35 | ± | 7.84 | 4.82 | ± | 0.03 | BD | ± | BD | 5.64 | ± | 1.43 |
|  |  | Pontederia | 1.61 | ± | 0.00 | BD | ± | BD | BD | ± | BD | BD | ± | BD |
| 19 | No Plant | Mat | 35.99 | ± | 7.66 | 4.54 | ± | 0.30 | 0.76 | ± | 0.07 | 6.44 | ± | 1.91 |
|  |  | No Mat | 19.73 | ± | 6.47 | 3.20 | ± | 0.18 | 0.68 | ± | 0.19 | 4.94 | ± | 1.43 |
|  | Plant + Mat | Juncus | 29.07 | ± | 9.95 | 3.12 | ± | 0.25 | BD | ± | BD | 7.04 | ± | 2.18 |
|  |  | Pontederia | 15.89 | ± | 3.15 | 1.20 | ± | 0.36 | BD | ± | BD | 8.59 | ± | 0.00 |

Notes: n=8, BD = below detection.
