# Supplementary material for: Data on floating treatment wetland aided nutrient removal from agricultural runoff using two wetland species
Source: Data Brief. 2018 Dec 15;22:756–61. doi: 10.1016/j.dib.2018.12.037 (PMC6330358; doi:10.1016/j.dib.2018.12.037)
Supplement: Supplementary file 2 — Summary water quality data of mesocosm experiment. [file mmc2.zip › Table A-7.docx]

**Table A-7.** Ion species concentration for potassium, sulfate, calcium, magnesium, and chloride at day 7 of 5 of 19 weeks.

| **Exper-** | **Presence or** | **Mat, No** |  |  |  |  |  |  |  |  |  |  |  |  |  |  |  |
| --- | --- | --- | --- | --- | --- | --- | --- | --- | --- | --- | --- | --- | --- | --- | --- | --- | --- |
| **Mental** | **absence** | **mat/taxa** |  |  |  |  |  |  |  |  |  |  |  |  |  |  |  |
| **Week No.** | **of plants** | **planted** |  |  |  |  |  |  |  |  |  |  |  |  |  |  |  |
|  |  | **within mat** | **Potassium (mg/L)** | | | **Sulfate (mg/L)** | | | **Calcium (mg/L)** | | | **Magnesium (mg/L)** | | | **Chloride (mg/L)** | | |
| 3 | No Plant | Mat | 6.67 | ± | 0.81 | 47.37 | ± | 3.34 | 13.61 | ± | 0.98 | 3.82 | ± | 0.33 | 17.00 | ± | 0.28 |
|  |  | No Mat | 6.75 | ± | 0.75 | 48.29 | ± | 2.36 | 14.01 | ± | 0.68 | 3.90 | ± | 0.27 | 17.74 | ± | 0.35 |
|  | Plant + Mat | Juncus | 6.32 | ± | 0.79 | 48.98 | ± | 3.26 | 13.89 | ± | 0.92 | 4.17 | ± | 0.38 | 17.12 | ± | 0.40 |
|  |  | Pontederia | 2.52 | ± | 0.36 | 44.90 | ± | 2.58 | 12.65 | ± | 0.67 | 3.52 | ± | 0.22 | 15.95 | ± | 0.21 |
| 7 | No Plant | Mat | 9.97 | ± | 1.71 | 53.04 | ± | 2.75 | 15.30 | ± | 0.56 | 4.37 | ± | 0.24 | 19.77 | ± | 0.30 |
|  |  | No Mat | 10.70 | ± | 1.84 | 54.49 | ± | 2.22 | 16.04 | ± | 0.46 | 4.66 | ± | 0.26 | 21.14 | ± | 0.46 |
|  | Plant + Mat | Juncus | 6.40 | ± | 1.99 | 54.94 | ± | 3.60 | 15.99 | ± | 0.63 | 4.55 | ± | 0.37 | 19.43 | ± | 0.50 |
|  |  | Pontederia | BD | ± | BD | 56.42 | ± | 3.92 | 12.51 | ± | 0.80 | 3.43 | ± | 0.18 | 10.09 | ± | 0.67 |
| 11 | No Plant | Mat | 9.43 | ± | 1.59 | 46.91 | ± | 1.69 | 12.62 | ± | 0.40 | 3.40 | ± | 0.05 | 17.37 | ± | 0.11 |
|  |  | No Mat | 9.63 | ± | 1.64 | 50.35 | ± | 1.60 | 13.57 | ± | 0.29 | 3.46 | ± | 0.09 | 18.74 | ± | 0.11 |
|  | Plant + Mat | Juncus | 6.20 | ± | 1.64 | 48.70 | ± | 1.41 | 14.34 | ± | 0.32 | 3.28 | ± | 0.09 | 17.18 | ± | 0.10 |
|  |  | Pontederia | BD | ± | BD | 51.53 | ± | 2.17 | 9.87 | ± | 0.35 | 1.85 | ± | 0.10 | 8.68 | ± | 0.56 |
| 15 | No Plant | Mat | 10.31 | ± | 1.74 | 49.67 | ± | 1.66 | 13.99 | ± | 0.21 | 3.58 | ± | 0.03 | 18.84 | ± | 0.06 |
|  |  | No Mat | 10.45 | ± | 1.84 | 51.31 | ± | 1.70 | 13.87 | ± | 0.09 | 3.48 | ± | 0.02 | 19.90 | ± | 0.16 |
|  | Plant + Mat | Juncus | 7.60 | ± | 2.22 | 51.61 | ± | 1.68 | 14.82 | ± | 0.12 | 3.46 | ± | 0.03 | 18.70 | ± | 0.26 |
|  |  | Pontederia | BD | ± | BD | 56.84 | ± | 2.35 | 10.59 | ± | 0.31 | 1.90 | ± | 0.07 | 8.55 | ± | 0.97 |
| 19 | No Plant | Mat | 12.09 | ± | 2.09 | 65.03 | ± | 5.02 | 17.90 | ± | 0.92 | 4.75 | ± | 0.30 | 23.55 | ± | 1.13 |
|  |  | No Mat | 11.48 | ± | 1.93 | 61.94 | ± | 3.63 | 17.08 | ± | 0.48 | 4.47 | ± | 0.18 | 22.88 | ± | 0.52 |
|  | Plant + Mat | Juncus | 8.52 | ± | 2.41 | 68.99 | ± | 5.21 | 19.37 | ± | 1.07 | 4.77 | ± | 0.25 | 23.94 | ± | 1.09 |
|  |  | Pontederia | 0.08 | ± | 0.08 | 68.56 | ± | 6.13 | 13.99 | ± | 0.66 | 3.58 | ± | 0.36 | 15.73 | ± | 2.28 |

Notes: n=8, BD = below detection.
