# Supplementary material for: Data on floating treatment wetland aided nutrient removal from agricultural runoff using two wetland species
Source: Data Brief. 2018 Dec 15;22:756–61. doi: 10.1016/j.dib.2018.12.037 (PMC6330358; doi:10.1016/j.dib.2018.12.037)
Supplement: Supplementary file 3 — Summary plant tissue data of mesocosm experiment. [file mmc3.zip › Table B-1.docx]

**Table B-1.** Root and shoot mineral nutrient concentrations for nitrogen, phosphorus, potassium, calcium, magnesium, and sulfur for two plant taxa grown in FTW and receiving high or low nutrient concentration weekly for 19 weeks

.

| **Plant taxa** | **Plant Part** | **Concentration (High/Low)** | **Nitrogen (%)** | | | | **Phosphorus (%)** | | | | **Potassium (%)** | | | | **Calcium (%)** | | | | **Magnesium (%)** | | | | **Sulfur (%)** | | | |
| --- | --- | --- | --- | --- | --- | --- | --- | --- | --- | --- | --- | --- | --- | --- | --- | --- | --- | --- | --- | --- | --- | --- | --- | --- | --- | --- |
| Juncus | Roots | Low | 1.510 | ± | 0.105 | 0.148 | | ± | 0.011 | 1.680 | | ± | 0.056 | 0.203 | | ± | 0.006 | 0.055 | | ± | 0.005 | 0.253 | | ± | 0.006 |  |
|  |  | High | 2.478 | ± | 0.136 | 0.445 | | ± | 0.010 | 1.865 | | ± | 0.150 | 0.135 | | ± | 0.009 | 0.060 | | ± | 0.000 | 0.468 | | ± | 0.009 |  |
|  | Shoots | Low | 1.808 | ± | 0.076 | 0.185 | | ± | 0.010 | 1.605 | | ± | 0.073 | 0.280 | | ± | 0.008 | 0.215 | | ± | 0.012 | 0.285 | | ± | 0.010 |  |
|  |  | High | 2.213 | ± | 0.053 | 0.298 | | ± | 0.011 | 1.725 | | ± | 0.020 | 0.225 | | ± | 0.006 | 0.180 | | ± | 0.015 | 0.335 | | ± | 0.003 |  |
| Pontederia | Roots | Low | 1.348 | ± | 0.014 | 0.105 | | ± | 0.006 | 0.720 | | ± | 0.029 | 0.468 | | ± | 0.025 | 0.075 | | ± | 0.005 | 0.285 | | ± | 0.022 |  |
|  |  | High | 1.920 | ± | 0.030 | 0.175 | | ± | 0.005 | 0.860 | | ± | 0.074 | 0.273 | | ± | 0.030 | 0.040 | | ± | 0.006 | 0.363 | | ± | 0.023 |  |
|  | Shoots | Low | 1.058 | ± | 0.027 | 0.108 | | ± | 0.003 | 1.270 | | ± | 0.007 | 1.175 | | ± | 0.031 | 0.288 | | ± | 0.014 | 0.185 | | ± | 0.006 |  |
|  |  | High | 1.930 | ± | 0.157 | 0.370 | | ± | 0.061 | 2.083 | | ± | 0.482 | 0.835 | | ± | 0.126 | 0.248 | | ± | 0.025 | 0.285 | | ± | 0.021 |  |

Note: n=4.
