# Supplementary material for: Data on floating treatment wetland aided nutrient removal from agricultural runoff using two wetland species
Source: Data Brief. 2018 Dec 15;22:756–61. doi: 10.1016/j.dib.2018.12.037 (PMC6330358; doi:10.1016/j.dib.2018.12.037)
Supplement: Supplementary file 3 — Summary plant tissue data of mesocosm experiment. [file mmc3.zip › Table B-2.docx]

**Table B-2.** Root and shoot mineral nutrient concentrations for zinc, copper, managanese, and

ironfor two plant taxa grown in FTW and receiving high or low nutrient concentration weekly for 19 weeks.

| **Plant taxa** | **Plant Pars** | **Concentration (High/Low)** | **Zinc (mg/kg)** | | | **Copper (mg/kg)** | | | **Manganese (mg/kg)** | | | **Iron (mg/kg)** | | |
| --- | --- | --- | --- | --- | --- | --- | --- | --- | --- | --- | --- | --- | --- | --- |
| Juncus | Roots | Low | 134.25 | ± | 36.0 | 41.00 | ± | 11.9 | 453.50 | ± | 175.4 | 296.00 | ± | 59.5 |
|  |  | High | 86.25 | ± | 16.8 | 200.50 | ± | 39.0 | 506.25 | ± | 99.4 | 713.50 | ± | 163.3 |
|  | Shoots | Low | 62.25 | ± | 4.3 | 21.00 | ± | 0.8 | 267.50 | ± | 14.8 | 113.75 | ± | 20.4 |
|  |  | High | 97.00 | ± | 7.9 | 28.00 | ± | 2.3 | 224.25 | ± | 14.4 | 173.50 | ± | 21.4 |
| Pontederia | Roots | Low | 39.50 | ± | 7.0 | 52.25 | ± | 4.4 | 31.00 | ± | 2.3 | 348.25 | ± | 14.3 |
|  |  | High | 52.25 | ± | 6.7 | 45.25 | ± | 6.1 | 32.50 | ± | 1.2 | 247.75 | ± | 29.7 |
|  | Shoots | Low | 48.50 | ± | 0.5 | 18.50 | ± | 0.5 | 188.25 | ± | 7.0 | 50.50 | ± | 1.0 |
|  |  | High | 82.00 | ± | 11.1 | 18.00 | ± | 1.8 | 127.25 | ± | 16.8 | 64.50 | ± | 4.5 |

Note: n=4.
