# Supplementary material for: Data on floating treatment wetland aided nutrient removal from agricultural runoff using two wetland species
Source: Data Brief. 2018 Dec 15;22:756–61. doi: 10.1016/j.dib.2018.12.037 (PMC6330358; doi:10.1016/j.dib.2018.12.037)
Supplement: Supplementary file 4 — Summary water temperature data. [file mmc4.zip › Table C-1.docx]

Table C-1. Daily minimum and maximum water temperatures in select mesocosms

|  | **M1-Pontederia** | | **M2-Juncus** | | **M8-No Mat** | | **M4-Mat** | |
| --- | --- | --- | --- | --- | --- | --- | --- | --- |
| **Date** | **Min** | **Max** | **Min** | **Max** | **Min** | **Max** | **Min** | **Max** |
|  | **(°C.)** | **(°C.)** | **(°C.)** | **(°C.)** | **(°C.)** | **(°C.)** | **(°C.)** | **(°C.)** |
| 6/15/2015 | 27 | 34 | 27 | 33.5 | 27 | 38.5 | 27 | 35 |
| 6/16/2015 | 30.5 | 36 | 30.5 | 35 | 29.5 | 38 | 31 | 36.5 |
| 6/17/2015 | 30.5 | 34 | 30 | 33.5 | 28 | 34.5 | 30 | 34 |
| 6/18/2015 | 27.5 | 32.5 | 27.5 | 32 | 25.5 | 35.5 | 27.5 | 32.5 |
| 6/19/2015 | 29 | 33.5 | 29 | 33 | 27.5 | 36.5 | 29 | 33.5 |
| 6/20/2015 | 30 | 33.5 | 29.5 | 32.5 | 27.5 | 35.5 | 30 | 33.5 |
| 6/21/2015 | 28.5 | 33 | 28 | 32 | 26 | 36 | 28 | 33 |
| 6/22/2015 | 30 | 34 | 29.5 | 34 | 28 | 37.5 | 29.5 | 35.5 |
| 6/23/2015 | 30 | 35.5 | 30 | 34.5 | 28.5 | 36.5 | 30.5 | 36 |
| 6/24/2015 | 29.5 | 34 | 29 | 33.5 | 27 | 33 | 29 | 34.5 |
| 6/25/2015 | 27.5 | 31.5 | 27.5 | 30.5 | 26 | 31.5 | 27.5 | 31 |
| 6/26/2015 | 25.5 | 29 | 25.5 | 29 | 23.5 | 28 | 25.5 | 29 |
| 6/27/2015 | 25 | 27 | 25 | 27 | 24 | 27 | 25 | 27 |
| 6/28/2015 | 25 | 29 | 25 | 28.5 | 24.5 | 30.5 | 25.5 | 29 |
| 6/29/2015 | 24 | 29.5 | 24.5 | 30 | 23 | 34 | 24.5 | 31 |
| 6/30/2015 | 26.5 | 32 | 27 | 31.5 | 26.5 | 33.5 | 27 | 32 |
| 7/1/2015 | 28 | 31.5 | 27.5 | 31 | 26 | 32 | 27.5 | 31.5 |
| 7/2/2015 | 28 | 30.5 | 28 | 30 | 26.5 | 30 | 28 | 30.5 |
| 7/3/2015 | 26 | 29.5 | 26 | 29 | 24.5 | 30 | 26 | 29.5 |
| 7/4/2015 | 26 | 30 | 26 | 29.5 | 25 | 31 | 26 | 30 |
| 7/5/2015 | 27 | 30 | 27 | 29.5 | 26 | 31 | 27 | 30 |
| 7/6/2015 | 26.5 | 30.5 | 26.5 | 30.5 | 26 | 32 | 27 | 31.5 |
| 7/7/2015 | 27 | 31.5 | 27 | 31 | 26 | 32 | 27.5 | 32 |
| 7/8/2015 | 28.5 | 31.5 | 28 | 31.5 | 27.5 | 31 | 28.5 | 31.5 |
| 7/9/2015 | 28.5 | 33 | 28.5 | 33 | 27.5 | 33.5 | 28.5 | 33 |
| 7/10/2015 | 30 | 33 | 30 | 32.5 | 28 | 34 | 30 | 33 |
| 7/11/2015 | 26 | 31.5 | 26 | 31 | 24.5 | 30.5 | 26 | 31.5 |
| 7/12/2015 | 25 | 28 | 25 | 28 | 23.5 | 29.5 | 25 | 28.5 |
| 7/13/2015 | 26 | 29.5 | 26 | 30 | 25.5 | 31 | 26 | 30.5 |
| 7/14/2015 | 26 | 29.5 | 26.5 | 29 | 25 | 29.5 | 26.5 | 29.5 |
| 7/15/2015 | 27 | 31.5 | 27 | 31 | 25.5 | 32 | 27 | 31 |
| 7/16/2015 | 26 | 30 | 26.5 | 30 | 25 | 29.5 | 26.5 | 30 |
| 7/17/2015 | 24.5 | 28.5 | 25 | 29 | 23 | 30 | 25 | 29 |
| 7/18/2015 | 26 | 30.5 | 26.5 | 31 | 25 | 31.5 | 26.5 | 30.5 |
| 7/19/2015 | 25.5 | 30.5 | 26 | 30.5 | 24.5 | 32.5 | 26 | 30.5 |
| 7/20/2015 | 28.5 | 33 | 28.5 | 33.5 | 27.5 | 35 | 28.5 | 34 |
| 7/21/2015 | 29.5 | 33.5 | 30 | 33.5 | 28.5 | 35 | 30 | 34 |
| 7/22/2015 | 28.5 | 31.5 | 29 | 32 | 27 | 33.5 | 29.5 | 32 |
| 7/23/2015 | 26 | 29.5 | 27.5 | 30 | 25.5 | 30.5 | 28 | 30.5 |
| 7/24/2015 | 26.5 | 30 | 27.5 | 30.5 | 26 | 32.5 | 27.5 | 31 |
| 7/25/2015 | 25 | 29 | 26.5 | 30 | 25 | 31.5 | 26.5 | 30.5 |
| 7/26/2015 | 24 | 29.5 | 25.5 | 30 | 24.5 | 32 | 25.5 | 30.5 |
| 7/27/2015 | 26.5 | 31 | 27.5 | 31.5 | 27 | 32 | 28 | 31.5 |
| 7/28/2015 | 27 | 31 | 28 | 31 | 27.5 | 32.5 | 28 | 32 |
| 7/29/2015 | 27 | 31.5 | 28.5 | 31.5 | 27.5 | 33 | 28.5 | 32.5 |
| 7/30/2015 | 27 | 32 | 28.5 | 32 | 27 | 33.5 | 28.5 | 33 |
| 7/31/2015 | 27.5 | 31 | 28.5 | 31.5 | 26.5 | 32.5 | 28.5 | 32 |
| 8/1/2015 | 25 | 30 | 27 | 30.5 | 25.5 | 31.5 | 27 | 31 |
| 8/2/2015 | 26.5 | 30.5 | 28 | 30.5 | 26 | 32 | 28 | 31 |
| 8/3/2015 | 26.5 | 32.5 | 27.5 | 33.5 | 26 | 36.5 | 28 | 34 |
| 8/4/2015 | 27 | 31.5 | 28.5 | 32.5 | 26 | 34 | 28.5 | 32.5 |
| 8/5/2015 | 27.5 | 31.5 | 28.5 | 32.5 | 26.5 | 34 | 29 | 33 |
| 8/6/2015 | 27 | 30 | 28.5 | 31.5 | 27 | 31.5 | 29 | 31.5 |
| 8/7/2015 | 25.5 | 27 | 26.5 | 29 | 25.5 | 28 | 26.5 | 29 |
| 8/8/2015 | 23.5 | 25 | 25 | 26.5 | 23.5 | 26.5 | 24.5 | 26.5 |
| 8/9/2015 | 22 | 26 | 23 | 27 | 21 | 29 | 23 | 27 |
| 8/10/2015 | 23 | 28 | 24.5 | 28.5 | 23.5 | 30.5 | 24.5 | 29 |
| 8/11/2015 | 24.5 | 26.5 | 25.5 | 27.5 | 24.5 | 27.5 | 26 | 28 |
| 8/12/2015 | 24 | 27.5 | 25 | 28 | 23.5 | 30.5 | 25 | 28 |
| 8/13/2015 | 23.5 | 27.5 | 25 | 28 | 24 | 30.5 | 25 | 28.5 |
| 8/14/2015 | 23.5 | 27.5 | 25 | 28.5 | 24 | 31 | 25 | 29 |
| 8/15/2015 | 22 | 26.5 | 24 | 27.5 | 22.5 | 30 | 24 | 28 |
| 8/16/2015 | 21.5 | 26.5 | 23.5 | 27.5 | 22 | 30.5 | 24 | 28.5 |
| 8/17/2015 | 23 | 30 | 24.5 | 30.5 | 23.5 | 35.5 | 25 | 31.5 |
| 8/18/2015 | 26 | 29 | 27 | 29.5 | 25.5 | 30.5 | 27.5 | 30 |
| 8/19/2015 | 26.5 | 29.5 | 27 | 30.5 | 26 | 31.5 | 27.5 | 31 |
| 8/20/2015 | 27 | 30 | 27.5 | 30 | 26.5 | 32.5 | 28 | 31 |
| 8/21/2015 | 26 | 28 | 27 | 29 | 26 | 30.5 | 27 | 29.5 |
| 8/22/2015 | 25 | 28 | 26 | 28.5 | 24 | 29.5 | 26 | 29 |
| 8/23/2015 | 24 | 27 | 25.5 | 28 | 23 | 29.5 | 25 | 28.5 |
| 8/24/2015 | 23 | 30.5 | 24.5 | 31 | 23 | 34 | 24.5 | 31.5 |
| 8/25/2015 | 26.5 | 29 | 27.5 | 29.5 | 25.5 | 31 | 27.5 | 30 |
| 8/26/2015 | 25.5 | 27 | 26.5 | 28.5 | 25.5 | 29 | 27 | 29 |
| 8/27/2015 | 23.5 | 26 | 25 | 27 | 22 | 27 | 24.5 | 27.5 |
| 8/28/2015 | 23 | 26 | 24 | 27 | 22.5 | 28.5 | 24 | 28 |
| 8/29/2015 | 21.5 | 26 | 23 | 26.5 | 22.5 | 28 | 23.5 | 27.5 |
| 8/30/2015 | 22 | 26.5 | 23.5 | 27 | 23 | 28 | 24 | 28 |
| 8/31/2015 | 25 | 27 | 26 | 27.5 | 25.5 | 27.5 | 26 | 27.5 |
| 9/1/2015 | 25 | 27.5 | 25 | 28 | 24.5 | 30.5 | 25.5 | 29 |
| 9/2/2015 | 25 | 29 | 25.5 | 29 | 26 | 30.5 | 26 | 30.5 |
| 9/3/2015 | 25 | 28.5 | 25.5 | 29 | 24.5 | 31 | 26 | 30 |
| 9/4/2015 | 25.5 | 29 | 26.5 | 29.5 | 26 | 31 | 27 | 30.5 |
| 9/5/2015 | 25.5 | 27.5 | 26.5 | 28 | 25 | 28.5 | 26.5 | 29 |
| 9/6/2015 | 24.5 | 25.5 | 25.5 | 26.5 | 24 | 25.5 | 25.5 | 26.5 |
| 9/7/2015 | 23 | 28 | 23.5 | 28.5 | 22 | 32 | 23.5 | 29.5 |
| 9/8/2015 | 24.5 | 26.5 | 25.5 | 27.5 | 25 | 28.5 | 26 | 28 |
| 9/9/2015 | 25.5 | 28.5 | 25.5 | 28 | 25.5 | 30 | 26 | 29.5 |
| 9/10/2015 | 26.5 | 28.5 | 27 | 29 | 26 | 30 | 27 | 30 |
| 9/11/2015 | 24.5 | 26.5 | 25.5 | 27.5 | 24 | 28.5 | 25.5 | 28 |
| 9/12/2015 | 24 | 26.5 | 25 | 27 | 24.5 | 27.5 | 25.5 | 27.5 |
| 9/13/2015 | 21.5 | 24.5 | 22.5 | 25 | 22 | 25 | 23 | 25.5 |
| 9/14/2015 | 19 | 24.5 | 19.5 | 25.5 | 18 | 29 | 19.5 | 26.5 |
| 9/15/2015 | 18.5 | 22.5 | 20 | 23.5 | 18.5 | 26.5 | 20 | 25 |
| 9/16/2015 | 19 | 23 | 20 | 23.5 | 19.5 | 25.5 | 20.5 | 25 |
| 9/17/2015 | 20 | 23 | 21 | 23.5 | 20.5 | 25.5 | 21.5 | 24.5 |
| 9/18/2015 | 20 | 24 | 20.5 | 24.5 | 20 | 26.5 | 21 | 25.5 |
| 9/19/2015 | 21.5 | 25.5 | 22.5 | 26 | 22.5 | 28 | 23 | 27.5 |
| 9/20/2015 | 22.5 | 25 | 23 | 25.5 | 23 | 27 | 23.5 | 26.5 |
| 9/21/2015 | 23 | 26 | 23.5 | 26.5 | 22.5 | 27 | 24 | 27 |
| 9/22/2015 | 22.5 | 23.5 | 23 | 24.5 | 21.5 | 23.5 | 23 | 24.5 |
| 9/23/2015 | 21.5 | 23 | 22 | 23.5 | 20.5 | 23.5 | 21.5 | 23.5 |
| 9/24/2015 | 21 | 23.5 | 21.5 | 23.5 | 19.5 | 24 | 21 | 24 |
| 9/25/2015 | 22 | 22.5 | 22.5 | 23 | 21 | 22.5 | 22.5 | 23.5 |
| 9/26/2015 | 20 | 22 | 21 | 22.5 | 20 | 21.5 | 20.5 | 22.5 |
| 9/27/2015 | 21 | 23.5 | 21.5 | 24 | 20.5 | 24.5 | 21 | 24 |
| 9/28/2015 | 23 | 25.5 | 23 | 25.5 | 22.5 | 27.5 | 23 | 26 |
| 9/29/2015 | 24 | 26 | 24.5 | 26 | 24 | 27 | 24.5 | 26.5 |
| 9/30/2015 | 23.5 | 25.5 | 24 | 25.5 | 23.5 | 26 | 24 | 26 |
| 10/1/2015 | 19.5 | 23.5 | 19.5 | 24 | 19 | 23.5 | 19.5 | 24 |
| 10/2/2015 | 18 | 19.5 | 18.5 | 19.5 | 18 | 19 | 18.5 | 19.5 |
| 10/3/2015 | 18.5 | 22 | 18.5 | 22 | 18 | 23 | 18.5 | 22.5 |
| 10/4/2015 | 19 | 22 | 19.5 | 22 | 19 | 23 | 19.5 | 22.5 |
| 10/5/2015 | 18 | 19.5 | 18 | 20 | 17.5 | 20.5 | 18 | 20 |
| 10/6/2015 | 17.5 | 19.5 | 18 | 19.5 | 16.5 | 22 | 17.5 | 20.5 |
| 10/7/2015 | 16.5 | 20 | 17 | 20.5 | 16 | 23.5 | 17 | 21.5 |
| 10/8/2015 | 17.5 | 21.5 | 18 | 22 | 17.5 | 25 | 18.5 | 23 |
| 10/9/2015 | 19.5 | 23.5 | 20 | 23.5 | 19 | 26.5 | 20 | 24.5 |
| 10/10/2015 | 18 | 23 | 18.5 | 23.5 | 17 | 23 | 18.5 | 24 |
| 10/11/2015 | 17 | 19 | 17.5 | 19.5 | 16 | 20.5 | 17.5 | 19.5 |
| 10/12/2015 | 16 | 22 | 16.5 | 22.5 | 15 | 25.5 | 16.5 | 23.5 |
| 10/13/2015 | 19.5 | 22 | 20 | 22.5 | 19.5 | 23.5 | 20 | 23 |
| 10/14/2015 | 19 | 22 | 19.5 | 22 | 18.5 | 23 | 19.5 | 23 |
| 10/15/2015 | 17 | 20 | 17.5 | 20.5 | 16.5 | 21.5 | 17.5 | 21.5 |
| 10/16/2015 | 14 | 18 | 14.5 | 18.5 | 13 | 18.5 | 14.5 | 19.5 |
| 10/17/2015 | 12 | 16 | 12.5 | 16.5 | 10.5 | 16.5 | 12 | 16.5 |
| 10/18/2015 | 11 | 14 | 11.5 | 15 | 9 | 14 | 11 | 15 |
| 10/19/2015 | 10 | 11 | 10.5 | 12 | 8.5 | 10.5 | 10 | 12 |
