# Supplementary material for: Data on floating treatment wetland aided nutrient removal from agricultural runoff using two wetland species
Source: Data Brief. 2018 Dec 15;22:756–61. doi: 10.1016/j.dib.2018.12.037 (PMC6330358; doi:10.1016/j.dib.2018.12.037)
Supplement: Supplementary file 5 — Nitrogen and phosphorus weekly removal curves. Figure D-1. Weekly TP removal curves for high initial concentration (17.13 ± 0.24 mg L−1 TN and 2.61 ± 0.04 mg L−1 TP) Pontederia cordata treatments from June 2015 through October 2015. Figure D-2. Weekly fitted TN removal curves for high initial concentration (17.13 ± 0.24 mg L−1 TN and 2.61 ± 0.04 mg L−1 TP) Juncus effusus treatments from June 2015 through October 2015. Figure D-3. Weekly fitted TP removal curves for high initial concentration (17.13 ± 0.24 mg L−1 TN and 2.61 ± 0.04 mg L−1 TP) Juncus effusus treatments from June 2015 through October 2015. Figure D-4. Weekly fitted TN removal curves for low initial concentration (5.22 mg L−1 TN and 0.52 mg L−1 TP) Pontederia cordata treatments from June 2015 through October 2015. Figure D-5. Weekly fitted TP removal curves for low initial concentration (5.22 mg L−1 TN and 0.52 mg L−1 TP) Pontederia cordata treatments from June 2015 through October 2015. Figure D-6. Weekly fitted TN removal curves for low initial concentration (5.22 mg L−1 TN and 0.52 mg L−1 TP) Juncus effusus treatments from June 2015 through October 2015. Figure D-7. Weekly fitted TP removal curves for low initial concentration (5.22 mg L−1 TN and 0.52 mg L−1 TP) Juncus effusus treatments from June 2015 through October 2015. Figure D-8. Weekly fitted TN removal curves by day for high initial concentration (17.13 ± 0.24 mg L−1 TN and 2.61 ± 0.04 mg L−1 TP) Pontederia cordata treatments from June 2015 through October 2015. [file mmc5.zip › Table D-3.docx]

Table D-3. Summary of fit for TN removal by day for high initial concentration (17.13±0.24 mg∙L^-1^ TN and 2.61±0.04 mg∙L^-1^ TP) *Juncus effusus* treatments in a floating wetland study conducted from June 2015 through October 2015.

| **Model** | **AICc** | **BIC** | **SSE** | **MSE** | **RMSE** | **R-Square** |
| --- | --- | --- | --- | --- | --- | --- |
| Mechanistic Growth | 261.767 | 74.106 | 1.017 | 0.113 | 0.336 | 0.965 |
