# Supplementary material for: Data on floating treatment wetland aided nutrient removal from agricultural runoff using two wetland species
Source: Data Brief. 2018 Dec 15;22:756–61. doi: 10.1016/j.dib.2018.12.037 (PMC6330358; doi:10.1016/j.dib.2018.12.037)
Supplement: Supplementary file 5 — Nitrogen and phosphorus weekly removal curves. Figure D-1. Weekly TP removal curves for high initial concentration (17.13 ± 0.24 mg L−1 TN and 2.61 ± 0.04 mg L−1 TP) Pontederia cordata treatments from June 2015 through October 2015. Figure D-2. Weekly fitted TN removal curves for high initial concentration (17.13 ± 0.24 mg L−1 TN and 2.61 ± 0.04 mg L−1 TP) Juncus effusus treatments from June 2015 through October 2015. Figure D-3. Weekly fitted TP removal curves for high initial concentration (17.13 ± 0.24 mg L−1 TN and 2.61 ± 0.04 mg L−1 TP) Juncus effusus treatments from June 2015 through October 2015. Figure D-4. Weekly fitted TN removal curves for low initial concentration (5.22 mg L−1 TN and 0.52 mg L−1 TP) Pontederia cordata treatments from June 2015 through October 2015. Figure D-5. Weekly fitted TP removal curves for low initial concentration (5.22 mg L−1 TN and 0.52 mg L−1 TP) Pontederia cordata treatments from June 2015 through October 2015. Figure D-6. Weekly fitted TN removal curves for low initial concentration (5.22 mg L−1 TN and 0.52 mg L−1 TP) Juncus effusus treatments from June 2015 through October 2015. Figure D-7. Weekly fitted TP removal curves for low initial concentration (5.22 mg L−1 TN and 0.52 mg L−1 TP) Juncus effusus treatments from June 2015 through October 2015. Figure D-8. Weekly fitted TN removal curves by day for high initial concentration (17.13 ± 0.24 mg L−1 TN and 2.61 ± 0.04 mg L−1 TP) Pontederia cordata treatments from June 2015 through October 2015. [file mmc5.zip › Table D-4.docx]

Table D-4. Nonlinear regression parameters for TN removal for high initial concentration (17.13±0.24 mg∙L^-1^ TN and 2.61±0.04 mg∙L^-1^ TP) *Juncus effusus* treatments in a floating wetland study conducted from June 2015 through October 2015.

| **Experiment Week** | **Asymptote** | **Scale** | **Growth Rate** |
| --- | --- | --- | --- |
| 3 | 2.70 | 1.01 | 0.13 |
| 5 | -0.56 | 1.09 | -0.13 |
| 7 | -0.27 | 1.41 | -0.25 |
| 9 | 2.86 | 1.01 | 0.26 |
| 11 | 4.10 | 1.01 | 0.21 |
| 13 | 2.78 | 1.04 | 0.25 |
| 15 | 2.02 | 1.00 | 0.34 |
| 17 | 6.25 | 1.01 | 0.08 |

Note: 19^th^ week omitted.
