# Supplementary material for: Data on floating treatment wetland aided nutrient removal from agricultural runoff using two wetland species
Source: Data Brief. 2018 Dec 15;22:756–61. doi: 10.1016/j.dib.2018.12.037 (PMC6330358; doi:10.1016/j.dib.2018.12.037)
Supplement: Supplementary file 5 — Nitrogen and phosphorus weekly removal curves. Figure D-1. Weekly TP removal curves for high initial concentration (17.13 ± 0.24 mg L−1 TN and 2.61 ± 0.04 mg L−1 TP) Pontederia cordata treatments from June 2015 through October 2015. Figure D-2. Weekly fitted TN removal curves for high initial concentration (17.13 ± 0.24 mg L−1 TN and 2.61 ± 0.04 mg L−1 TP) Juncus effusus treatments from June 2015 through October 2015. Figure D-3. Weekly fitted TP removal curves for high initial concentration (17.13 ± 0.24 mg L−1 TN and 2.61 ± 0.04 mg L−1 TP) Juncus effusus treatments from June 2015 through October 2015. Figure D-4. Weekly fitted TN removal curves for low initial concentration (5.22 mg L−1 TN and 0.52 mg L−1 TP) Pontederia cordata treatments from June 2015 through October 2015. Figure D-5. Weekly fitted TP removal curves for low initial concentration (5.22 mg L−1 TN and 0.52 mg L−1 TP) Pontederia cordata treatments from June 2015 through October 2015. Figure D-6. Weekly fitted TN removal curves for low initial concentration (5.22 mg L−1 TN and 0.52 mg L−1 TP) Juncus effusus treatments from June 2015 through October 2015. Figure D-7. Weekly fitted TP removal curves for low initial concentration (5.22 mg L−1 TN and 0.52 mg L−1 TP) Juncus effusus treatments from June 2015 through October 2015. Figure D-8. Weekly fitted TN removal curves by day for high initial concentration (17.13 ± 0.24 mg L−1 TN and 2.61 ± 0.04 mg L−1 TP) Pontederia cordata treatments from June 2015 through October 2015. [file mmc5.zip › Table D-6.docx]

Table D-6. Nonlinear regression parameters for TP removal by high initial concentration (17.13±0.24 mg∙L^-1^ TN and 2.61±0.04 mg∙L^-1^ TP) *Juncus effusus* treatments in a floating wetland study conducted from June 2015 through October 2015.

| **Experiment Week** | **Asymptote** | **Scale** | **Growth Rate** |
| --- | --- | --- | --- |
| 3 | 0.760 | 1.020 | 0.078 |
| 5 | 0.003 | -0.500 | -0.585 |
| 7 | -0.005 | 0.013 | -1.192 |
| 9 | 0.081 | 1.131 | 0.462 |
| 11 | 0.507 | 1.021 | 0.099 |
| 13 | 0.474 | 1.065 | 0.236 |
| 15 | 0.164 | 1.000 | 17.015 |
| 17 | -0.060 | 0.462 | -0.375 |
| 19 | 0.106 | 0.988 | 0.611 |
