# Supplementary material for: Data on floating treatment wetland aided nutrient removal from agricultural runoff using two wetland species
Source: Data Brief. 2018 Dec 15;22:756–61. doi: 10.1016/j.dib.2018.12.037 (PMC6330358; doi:10.1016/j.dib.2018.12.037)
Supplement: Supplementary file 5 — Nitrogen and phosphorus weekly removal curves. Figure D-1. Weekly TP removal curves for high initial concentration (17.13 ± 0.24 mg L−1 TN and 2.61 ± 0.04 mg L−1 TP) Pontederia cordata treatments from June 2015 through October 2015. Figure D-2. Weekly fitted TN removal curves for high initial concentration (17.13 ± 0.24 mg L−1 TN and 2.61 ± 0.04 mg L−1 TP) Juncus effusus treatments from June 2015 through October 2015. Figure D-3. Weekly fitted TP removal curves for high initial concentration (17.13 ± 0.24 mg L−1 TN and 2.61 ± 0.04 mg L−1 TP) Juncus effusus treatments from June 2015 through October 2015. Figure D-4. Weekly fitted TN removal curves for low initial concentration (5.22 mg L−1 TN and 0.52 mg L−1 TP) Pontederia cordata treatments from June 2015 through October 2015. Figure D-5. Weekly fitted TP removal curves for low initial concentration (5.22 mg L−1 TN and 0.52 mg L−1 TP) Pontederia cordata treatments from June 2015 through October 2015. Figure D-6. Weekly fitted TN removal curves for low initial concentration (5.22 mg L−1 TN and 0.52 mg L−1 TP) Juncus effusus treatments from June 2015 through October 2015. Figure D-7. Weekly fitted TP removal curves for low initial concentration (5.22 mg L−1 TN and 0.52 mg L−1 TP) Juncus effusus treatments from June 2015 through October 2015. Figure D-8. Weekly fitted TN removal curves by day for high initial concentration (17.13 ± 0.24 mg L−1 TN and 2.61 ± 0.04 mg L−1 TP) Pontederia cordata treatments from June 2015 through October 2015. [file mmc5.zip › Table D-8.docx]

Table D-8. Nonlinear regression parameters for TN removal by low initial concentration (5.22 mg∙L^-1^ TN and 0.52 mg∙L^-1^ TP) *Pontederia cordata* treatments in a floating wetland study conducted from June 2015 through October 2015.

| **Experiment Week** | **Asymptote** | **Scale** | **Growth Rate** |
| --- | --- | --- | --- |
| 3 | 2.058 | 1.009 | 0.142 |
| 5 | 1.453 | 1.007 | 0.211 |
| 7 | 1.547 | 1.000 | 0.963 |
| 9 | 1.607 | 1.000 | 12.307 |
| 11 | 1.490 | 1.000 | 16.374 |
| 13 | 1.514 | 1.000 | 2.281 |
| 15 | 1.545 | 1.000 | 2.083 |
| 17 | 1.471 | 1.000 | 16.746 |
| 19 | 1.476 | 1.000 | 28.571 |
