# Supplementary figures and images for: Data on floating treatment wetland aided nutrient removal from agricultural runoff using two wetland species
Source: Data Brief. 2018 Dec 15;22:756–61. doi: 10.1016/j.dib.2018.12.037 (PMC6330358; doi:10.1016/j.dib.2018.12.037)

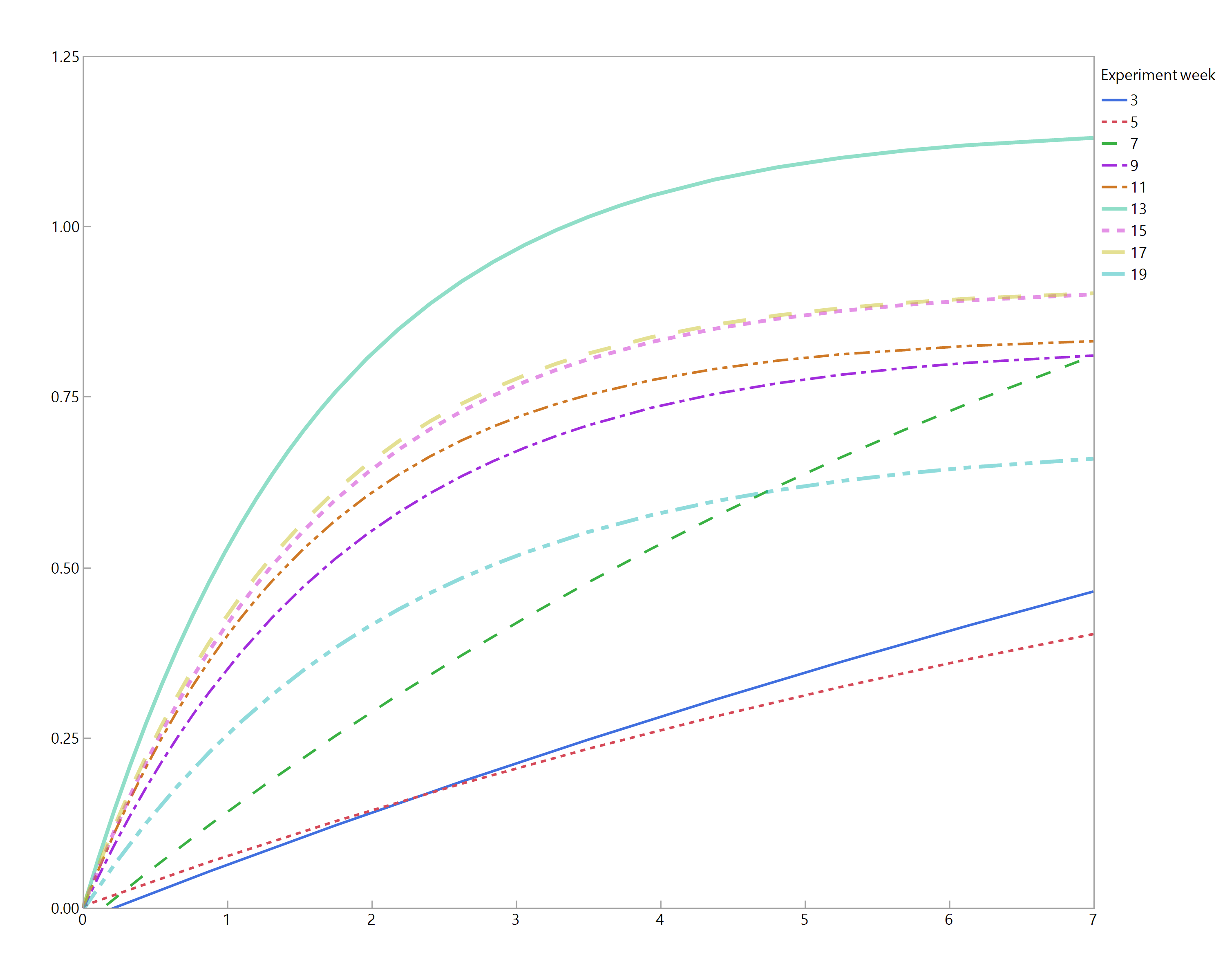

Supplement: Supplementary file 5 — Nitrogen and phosphorus weekly removal curves. Figure D-1. Weekly TP removal curves for high initial concentration (17.13 ± 0.24 mg L−1 TN and 2.61 ± 0.04 mg L−1 TP) Pontederia cordata treatments from June 2015 through October 2015. Figure D-2. Weekly fitted TN removal curves for high initial concentration (17.13 ± 0.24 mg L−1 TN and 2.61 ± 0.04 mg L−1 TP) Juncus effusus treatments from June 2015 through October 2015. Figure D-3. Weekly fitted TP removal curves for high initial concentration (17.13 ± 0.24 mg L−1 TN and 2.61 ± 0.04 mg L−1 TP) Juncus effusus treatments from June 2015 through October 2015. Figure D-4. Weekly fitted TN removal curves for low initial concentration (5.22 mg L−1 TN and 0.52 mg L−1 TP) Pontederia cordata treatments from June 2015 through October 2015. Figure D-5. Weekly fitted TP removal curves for low initial concentration (5.22 mg L−1 TN and 0.52 mg L−1 TP) Pontederia cordata treatments from June 2015 through October 2015. Figure D-6. Weekly fitted TN removal curves for low initial concentration (5.22 mg L−1 TN and 0.52 mg L−1 TP) Juncus effusus treatments from June 2015 through October 2015. Figure D-7. Weekly fitted TP removal curves for low initial concentration (5.22 mg L−1 TN and 0.52 mg L−1 TP) Juncus effusus treatments from June 2015 through October 2015. Figure D-8. Weekly fitted TN removal curves by day for high initial concentration (17.13 ± 0.24 mg L−1 TN and 2.61 ± 0.04 mg L−1 TP) Pontederia cordata treatments from June 2015 through October 2015. [file mmc5.zip › Figure D-1.tiff]

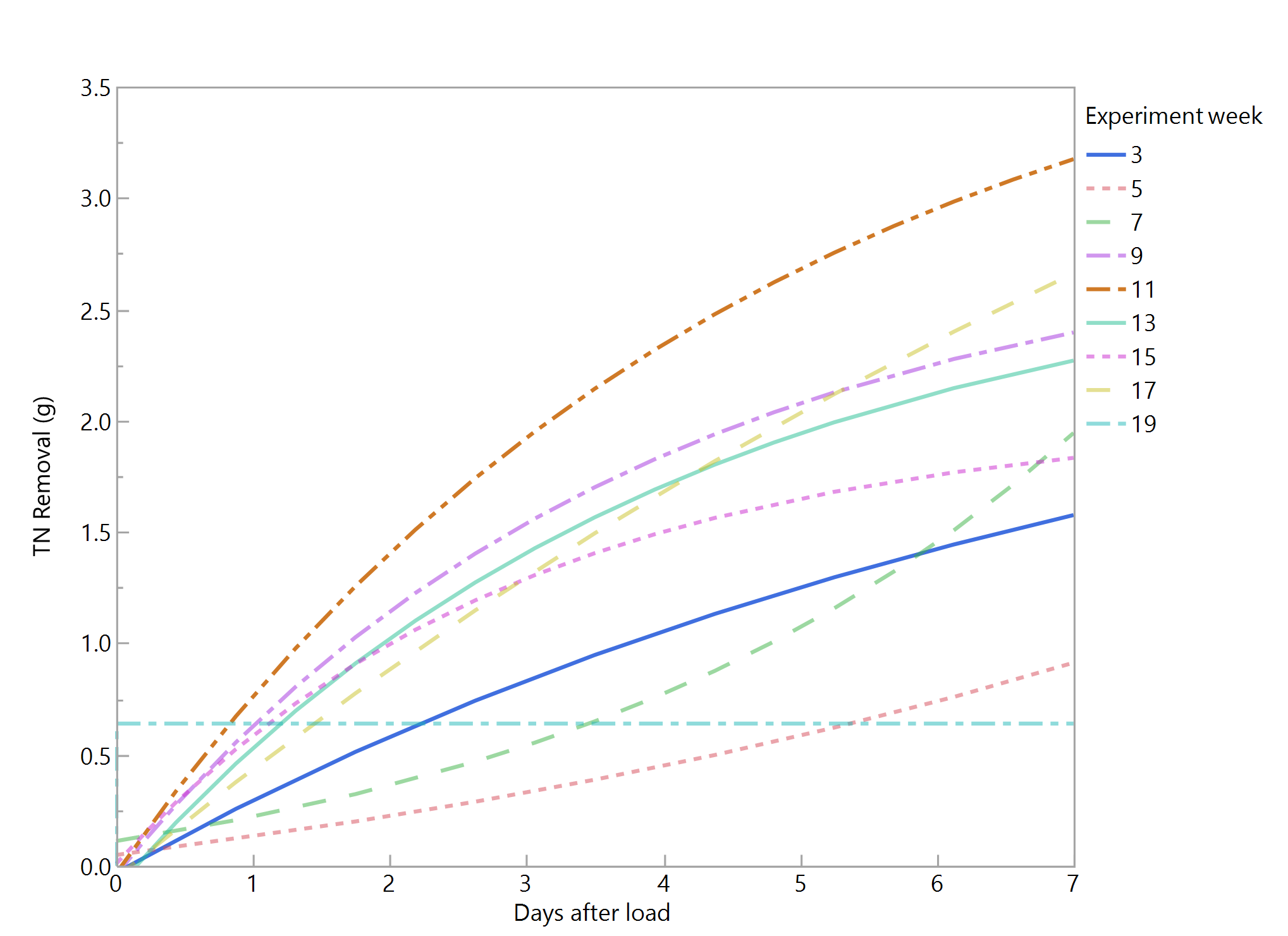

Supplement: Supplementary file 5 — Nitrogen and phosphorus weekly removal curves. Figure D-1. Weekly TP removal curves for high initial concentration (17.13 ± 0.24 mg L−1 TN and 2.61 ± 0.04 mg L−1 TP) Pontederia cordata treatments from June 2015 through October 2015. Figure D-2. Weekly fitted TN removal curves for high initial concentration (17.13 ± 0.24 mg L−1 TN and 2.61 ± 0.04 mg L−1 TP) Juncus effusus treatments from June 2015 through October 2015. Figure D-3. Weekly fitted TP removal curves for high initial concentration (17.13 ± 0.24 mg L−1 TN and 2.61 ± 0.04 mg L−1 TP) Juncus effusus treatments from June 2015 through October 2015. Figure D-4. Weekly fitted TN removal curves for low initial concentration (5.22 mg L−1 TN and 0.52 mg L−1 TP) Pontederia cordata treatments from June 2015 through October 2015. Figure D-5. Weekly fitted TP removal curves for low initial concentration (5.22 mg L−1 TN and 0.52 mg L−1 TP) Pontederia cordata treatments from June 2015 through October 2015. Figure D-6. Weekly fitted TN removal curves for low initial concentration (5.22 mg L−1 TN and 0.52 mg L−1 TP) Juncus effusus treatments from June 2015 through October 2015. Figure D-7. Weekly fitted TP removal curves for low initial concentration (5.22 mg L−1 TN and 0.52 mg L−1 TP) Juncus effusus treatments from June 2015 through October 2015. Figure D-8. Weekly fitted TN removal curves by day for high initial concentration (17.13 ± 0.24 mg L−1 TN and 2.61 ± 0.04 mg L−1 TP) Pontederia cordata treatments from June 2015 through October 2015. [file mmc5.zip › Figure D-2.tiff]

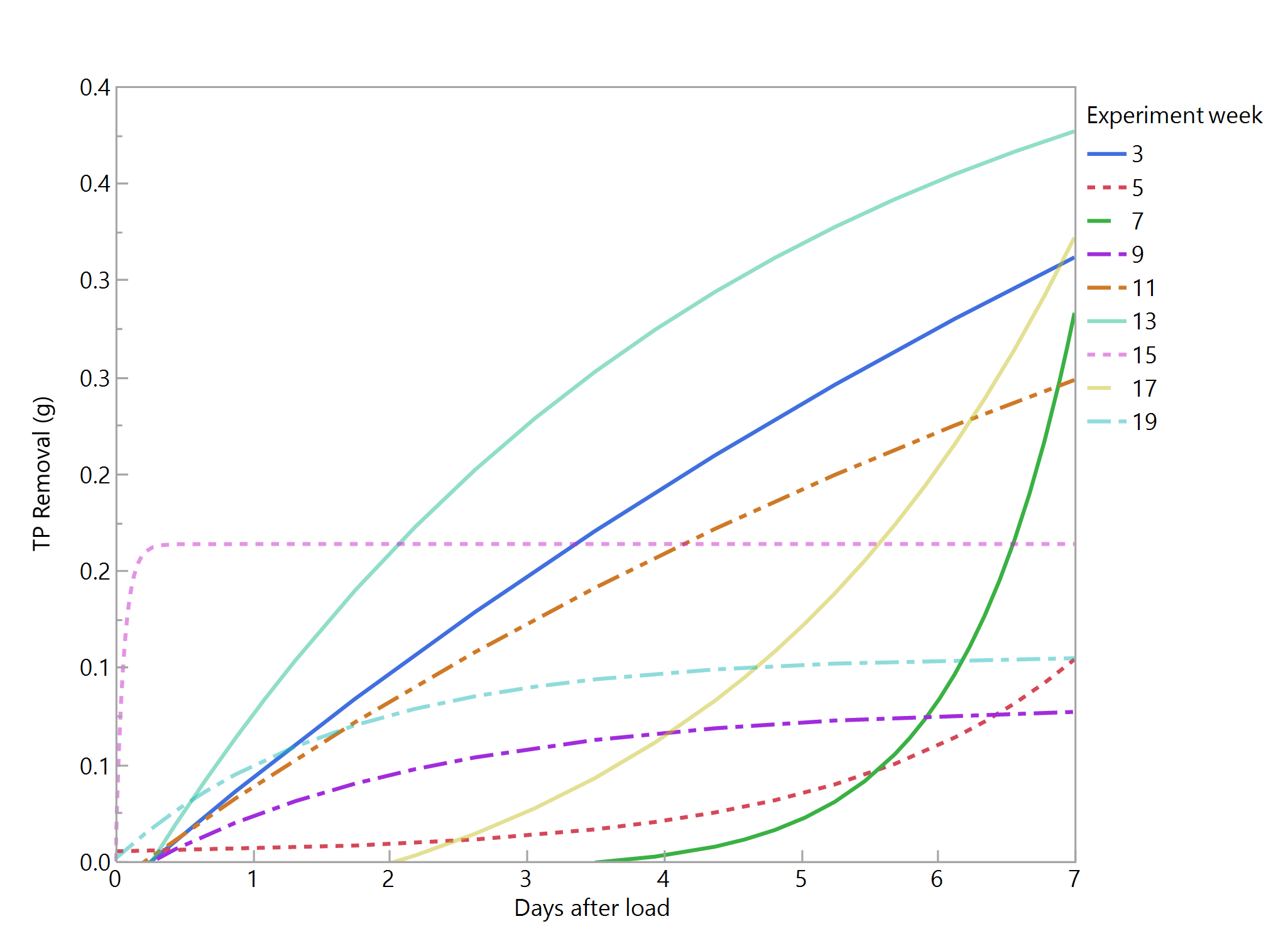

Supplement: Supplementary file 5 — Nitrogen and phosphorus weekly removal curves. Figure D-1. Weekly TP removal curves for high initial concentration (17.13 ± 0.24 mg L−1 TN and 2.61 ± 0.04 mg L−1 TP) Pontederia cordata treatments from June 2015 through October 2015. Figure D-2. Weekly fitted TN removal curves for high initial concentration (17.13 ± 0.24 mg L−1 TN and 2.61 ± 0.04 mg L−1 TP) Juncus effusus treatments from June 2015 through October 2015. Figure D-3. Weekly fitted TP removal curves for high initial concentration (17.13 ± 0.24 mg L−1 TN and 2.61 ± 0.04 mg L−1 TP) Juncus effusus treatments from June 2015 through October 2015. Figure D-4. Weekly fitted TN removal curves for low initial concentration (5.22 mg L−1 TN and 0.52 mg L−1 TP) Pontederia cordata treatments from June 2015 through October 2015. Figure D-5. Weekly fitted TP removal curves for low initial concentration (5.22 mg L−1 TN and 0.52 mg L−1 TP) Pontederia cordata treatments from June 2015 through October 2015. Figure D-6. Weekly fitted TN removal curves for low initial concentration (5.22 mg L−1 TN and 0.52 mg L−1 TP) Juncus effusus treatments from June 2015 through October 2015. Figure D-7. Weekly fitted TP removal curves for low initial concentration (5.22 mg L−1 TN and 0.52 mg L−1 TP) Juncus effusus treatments from June 2015 through October 2015. Figure D-8. Weekly fitted TN removal curves by day for high initial concentration (17.13 ± 0.24 mg L−1 TN and 2.61 ± 0.04 mg L−1 TP) Pontederia cordata treatments from June 2015 through October 2015. [file mmc5.zip › Figure D-3.tiff]

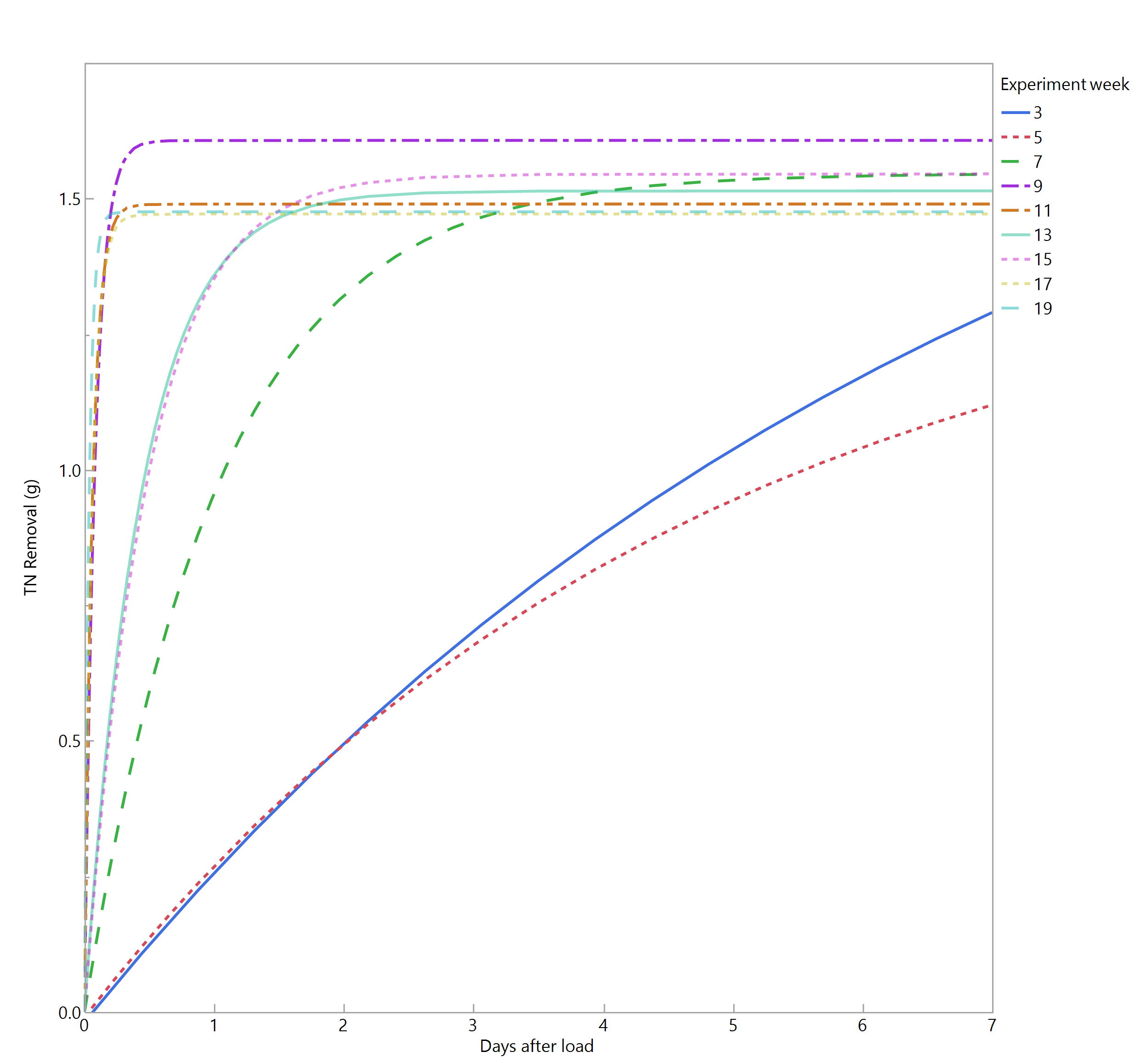

Supplement: Supplementary file 5 — Nitrogen and phosphorus weekly removal curves. Figure D-1. Weekly TP removal curves for high initial concentration (17.13 ± 0.24 mg L−1 TN and 2.61 ± 0.04 mg L−1 TP) Pontederia cordata treatments from June 2015 through October 2015. Figure D-2. Weekly fitted TN removal curves for high initial concentration (17.13 ± 0.24 mg L−1 TN and 2.61 ± 0.04 mg L−1 TP) Juncus effusus treatments from June 2015 through October 2015. Figure D-3. Weekly fitted TP removal curves for high initial concentration (17.13 ± 0.24 mg L−1 TN and 2.61 ± 0.04 mg L−1 TP) Juncus effusus treatments from June 2015 through October 2015. Figure D-4. Weekly fitted TN removal curves for low initial concentration (5.22 mg L−1 TN and 0.52 mg L−1 TP) Pontederia cordata treatments from June 2015 through October 2015. Figure D-5. Weekly fitted TP removal curves for low initial concentration (5.22 mg L−1 TN and 0.52 mg L−1 TP) Pontederia cordata treatments from June 2015 through October 2015. Figure D-6. Weekly fitted TN removal curves for low initial concentration (5.22 mg L−1 TN and 0.52 mg L−1 TP) Juncus effusus treatments from June 2015 through October 2015. Figure D-7. Weekly fitted TP removal curves for low initial concentration (5.22 mg L−1 TN and 0.52 mg L−1 TP) Juncus effusus treatments from June 2015 through October 2015. Figure D-8. Weekly fitted TN removal curves by day for high initial concentration (17.13 ± 0.24 mg L−1 TN and 2.61 ± 0.04 mg L−1 TP) Pontederia cordata treatments from June 2015 through October 2015. [file mmc5.zip › Figure D-4.tiff]

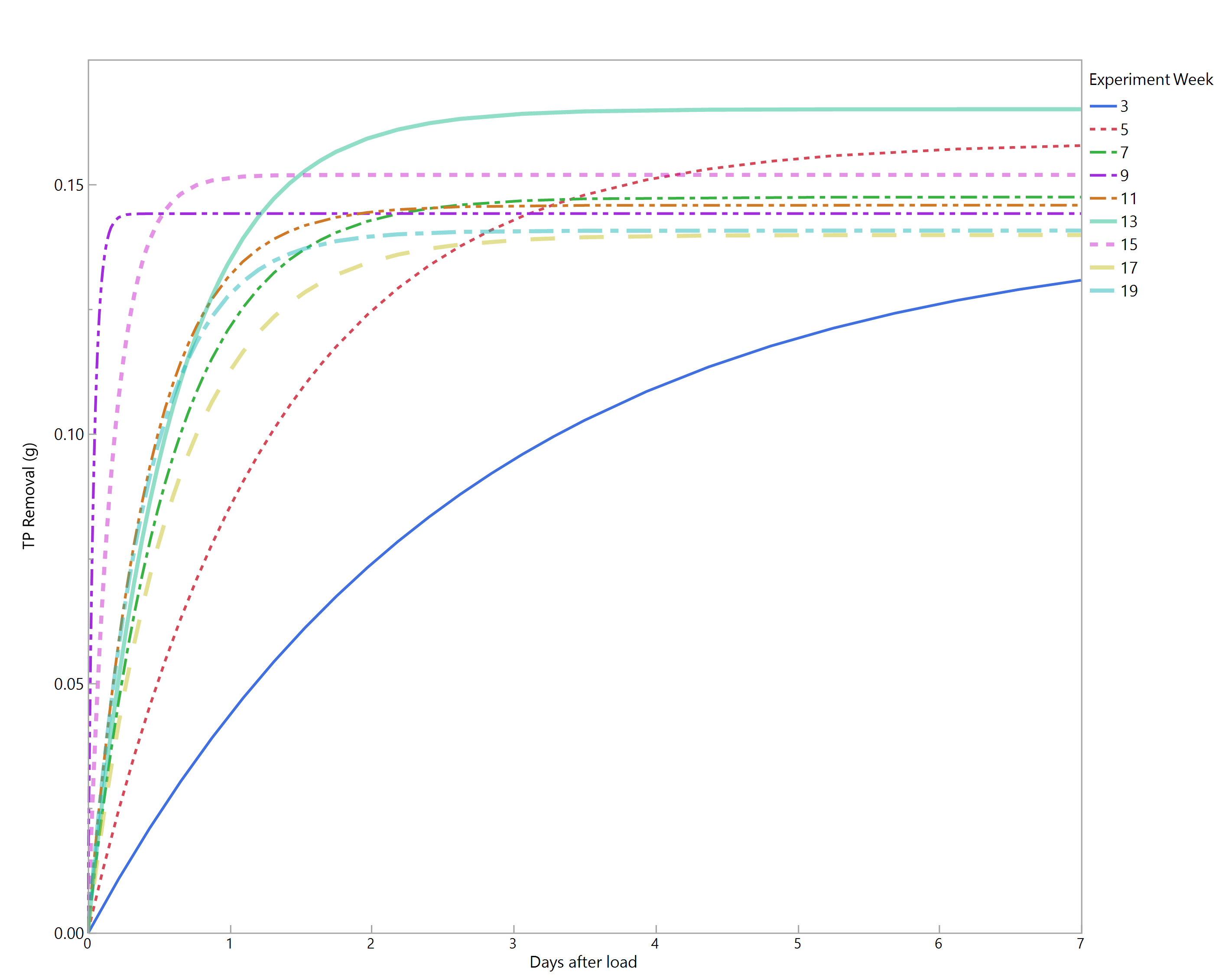

Supplement: Supplementary file 5 — Nitrogen and phosphorus weekly removal curves. Figure D-1. Weekly TP removal curves for high initial concentration (17.13 ± 0.24 mg L−1 TN and 2.61 ± 0.04 mg L−1 TP) Pontederia cordata treatments from June 2015 through October 2015. Figure D-2. Weekly fitted TN removal curves for high initial concentration (17.13 ± 0.24 mg L−1 TN and 2.61 ± 0.04 mg L−1 TP) Juncus effusus treatments from June 2015 through October 2015. Figure D-3. Weekly fitted TP removal curves for high initial concentration (17.13 ± 0.24 mg L−1 TN and 2.61 ± 0.04 mg L−1 TP) Juncus effusus treatments from June 2015 through October 2015. Figure D-4. Weekly fitted TN removal curves for low initial concentration (5.22 mg L−1 TN and 0.52 mg L−1 TP) Pontederia cordata treatments from June 2015 through October 2015. Figure D-5. Weekly fitted TP removal curves for low initial concentration (5.22 mg L−1 TN and 0.52 mg L−1 TP) Pontederia cordata treatments from June 2015 through October 2015. Figure D-6. Weekly fitted TN removal curves for low initial concentration (5.22 mg L−1 TN and 0.52 mg L−1 TP) Juncus effusus treatments from June 2015 through October 2015. Figure D-7. Weekly fitted TP removal curves for low initial concentration (5.22 mg L−1 TN and 0.52 mg L−1 TP) Juncus effusus treatments from June 2015 through October 2015. Figure D-8. Weekly fitted TN removal curves by day for high initial concentration (17.13 ± 0.24 mg L−1 TN and 2.61 ± 0.04 mg L−1 TP) Pontederia cordata treatments from June 2015 through October 2015. [file mmc5.zip › Figure D-5.tiff]

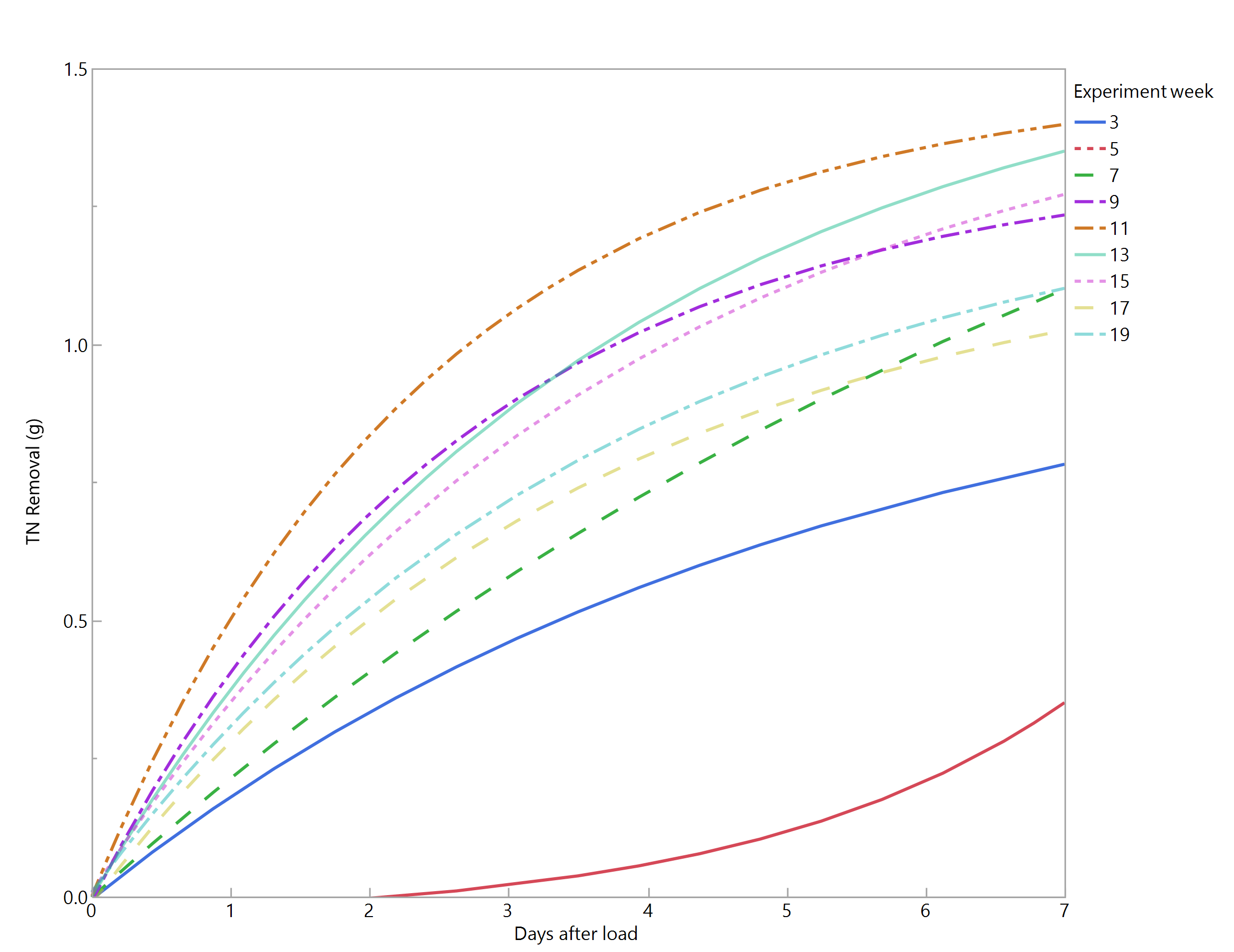

Supplement: Supplementary file 5 — Nitrogen and phosphorus weekly removal curves. Figure D-1. Weekly TP removal curves for high initial concentration (17.13 ± 0.24 mg L−1 TN and 2.61 ± 0.04 mg L−1 TP) Pontederia cordata treatments from June 2015 through October 2015. Figure D-2. Weekly fitted TN removal curves for high initial concentration (17.13 ± 0.24 mg L−1 TN and 2.61 ± 0.04 mg L−1 TP) Juncus effusus treatments from June 2015 through October 2015. Figure D-3. Weekly fitted TP removal curves for high initial concentration (17.13 ± 0.24 mg L−1 TN and 2.61 ± 0.04 mg L−1 TP) Juncus effusus treatments from June 2015 through October 2015. Figure D-4. Weekly fitted TN removal curves for low initial concentration (5.22 mg L−1 TN and 0.52 mg L−1 TP) Pontederia cordata treatments from June 2015 through October 2015. Figure D-5. Weekly fitted TP removal curves for low initial concentration (5.22 mg L−1 TN and 0.52 mg L−1 TP) Pontederia cordata treatments from June 2015 through October 2015. Figure D-6. Weekly fitted TN removal curves for low initial concentration (5.22 mg L−1 TN and 0.52 mg L−1 TP) Juncus effusus treatments from June 2015 through October 2015. Figure D-7. Weekly fitted TP removal curves for low initial concentration (5.22 mg L−1 TN and 0.52 mg L−1 TP) Juncus effusus treatments from June 2015 through October 2015. Figure D-8. Weekly fitted TN removal curves by day for high initial concentration (17.13 ± 0.24 mg L−1 TN and 2.61 ± 0.04 mg L−1 TP) Pontederia cordata treatments from June 2015 through October 2015. [file mmc5.zip › Figure D-6.tiff]

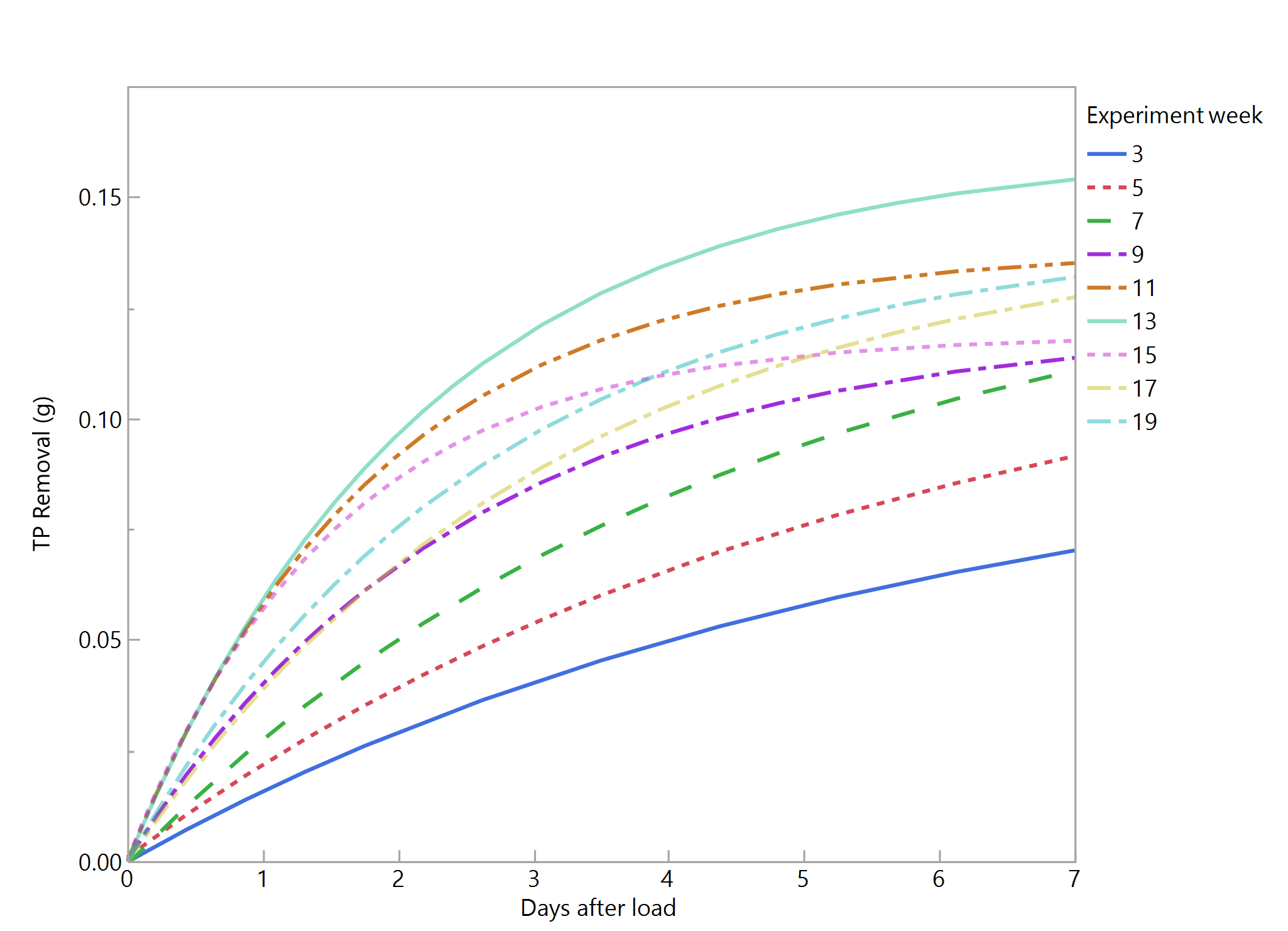

Supplement: Supplementary file 5 — Nitrogen and phosphorus weekly removal curves. Figure D-1. Weekly TP removal curves for high initial concentration (17.13 ± 0.24 mg L−1 TN and 2.61 ± 0.04 mg L−1 TP) Pontederia cordata treatments from June 2015 through October 2015. Figure D-2. Weekly fitted TN removal curves for high initial concentration (17.13 ± 0.24 mg L−1 TN and 2.61 ± 0.04 mg L−1 TP) Juncus effusus treatments from June 2015 through October 2015. Figure D-3. Weekly fitted TP removal curves for high initial concentration (17.13 ± 0.24 mg L−1 TN and 2.61 ± 0.04 mg L−1 TP) Juncus effusus treatments from June 2015 through October 2015. Figure D-4. Weekly fitted TN removal curves for low initial concentration (5.22 mg L−1 TN and 0.52 mg L−1 TP) Pontederia cordata treatments from June 2015 through October 2015. Figure D-5. Weekly fitted TP removal curves for low initial concentration (5.22 mg L−1 TN and 0.52 mg L−1 TP) Pontederia cordata treatments from June 2015 through October 2015. Figure D-6. Weekly fitted TN removal curves for low initial concentration (5.22 mg L−1 TN and 0.52 mg L−1 TP) Juncus effusus treatments from June 2015 through October 2015. Figure D-7. Weekly fitted TP removal curves for low initial concentration (5.22 mg L−1 TN and 0.52 mg L−1 TP) Juncus effusus treatments from June 2015 through October 2015. Figure D-8. Weekly fitted TN removal curves by day for high initial concentration (17.13 ± 0.24 mg L−1 TN and 2.61 ± 0.04 mg L−1 TP) Pontederia cordata treatments from June 2015 through October 2015. [file mmc5.zip › Figure D-7.tiff]

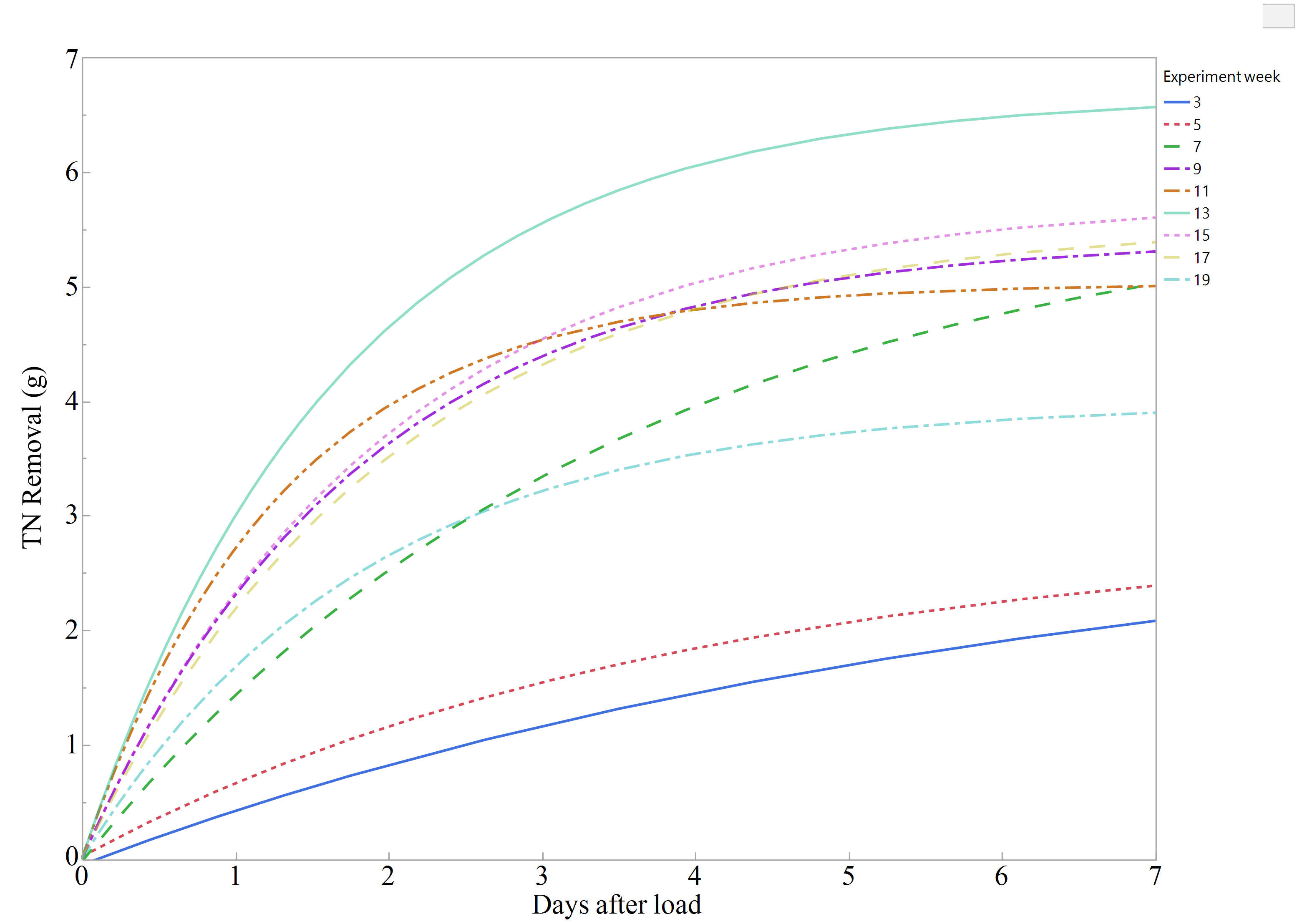

Supplement: Supplementary file 5 — Nitrogen and phosphorus weekly removal curves. Figure D-1. Weekly TP removal curves for high initial concentration (17.13 ± 0.24 mg L−1 TN and 2.61 ± 0.04 mg L−1 TP) Pontederia cordata treatments from June 2015 through October 2015. Figure D-2. Weekly fitted TN removal curves for high initial concentration (17.13 ± 0.24 mg L−1 TN and 2.61 ± 0.04 mg L−1 TP) Juncus effusus treatments from June 2015 through October 2015. Figure D-3. Weekly fitted TP removal curves for high initial concentration (17.13 ± 0.24 mg L−1 TN and 2.61 ± 0.04 mg L−1 TP) Juncus effusus treatments from June 2015 through October 2015. Figure D-4. Weekly fitted TN removal curves for low initial concentration (5.22 mg L−1 TN and 0.52 mg L−1 TP) Pontederia cordata treatments from June 2015 through October 2015. Figure D-5. Weekly fitted TP removal curves for low initial concentration (5.22 mg L−1 TN and 0.52 mg L−1 TP) Pontederia cordata treatments from June 2015 through October 2015. Figure D-6. Weekly fitted TN removal curves for low initial concentration (5.22 mg L−1 TN and 0.52 mg L−1 TP) Juncus effusus treatments from June 2015 through October 2015. Figure D-7. Weekly fitted TP removal curves for low initial concentration (5.22 mg L−1 TN and 0.52 mg L−1 TP) Juncus effusus treatments from June 2015 through October 2015. Figure D-8. Weekly fitted TN removal curves by day for high initial concentration (17.13 ± 0.24 mg L−1 TN and 2.61 ± 0.04 mg L−1 TP) Pontederia cordata treatments from June 2015 through October 2015. [file mmc5.zip › Figure D-8.tiff]
